# Global Prevalence of Alloimmunization in Adults with Sickle Cell Disease Receiving Red Blood Cell Transfusions: A Systematic Review and Meta-Analysis

**DOI:** 10.3390/jcm15103828

**Published:** 2026-05-15

**Authors:** Mortadah Alsalman, Jawad S. Alnajjar, Sarra Riyadh Alhassan, Hussain A. Almarzoug, Qusai A. Alobaid, Reham Riyadh Alhassan, Maryam Mohammed Alshams, Bdoor Abdulaziz Almoqren, Nabeel Baqer Al Besher, Abdullah Almaqhawi

**Affiliations:** 1 Department of Medicine, College of Medicine, King Faisal University, Al Ahsa 31982, Saudi Arabia; 2 College of Medicine, King Faisal University, Al Ahsa 31982, Saudi Arabia; jawd.alnajjar@gmail.com (J.S.A.); hussain_2022@hotmail.com (H.A.A.); qusay1423@gmail.com (Q.A.A.);; 3 Department of Laboratory and Blood Bank, Prince Saud bin Jalawi Hospital, Al Ahsa 36377, Saudi Arabia; nalbesher@hotmail.com; 4 Department of Family and Community Medicine, College of Medicine, King Faisal University, Al Ahsa 31982, Saudi Arabia; aalmuqahwi@kfu.edu.sa

**Keywords:** sickle cell anemia, blood transfusion, alloimmunization, isoantibodies, Rh-Hr blood-group system, Kell blood-group system, prevalence, meta-analysis

## Abstract

**Background/Objectives**: Blood transfusion is a crucial component in the treatment of individuals with sickle cell disease [SCD]; nonetheless, multiple transfusions can lead to considerable complications, notably alloimmunization. However, the prevalence of alloimmunization and its predictors remain incompletely explained. This review aimed to determine its global prevalence and identify associated risk factors. **Method**: Our protocol was registered in PROSPERO [ID: CRD420251167042] in accordance with the PRISMA 2020 criteria. A thorough literature search was conducted across PubMed, Embase, Web of Science, Scopus, and the Cochrane Library to identify studies reporting the prevalence of alloimmunization in adults with confirmed sickle cell disease who have received blood transfusions. This search included all publications up to 16 April 2026. Two reviewers independently screened and extracted data, and the Newcastle–Ottawa Scale was used to evaluate the study’s quality. After the Freeman–Tukey transformation, a random-effects model was used to estimate the pooled prevalence. We examined disparities among groups and geographies, study designs, and matching procedures to determine their differences. We additionally employed meta-regression to identify potential predictors. **Results**: Nine studies [n = 1711; 1978–2026] met the inclusion criteria. The overall rate of alloimmunization was 28.9% [95% CI 22.4–35.4; I^2^ = 88.5%]. The most prevalent antibodies were those of the Rh and Kell systems, with anti-E antibodies being the most frequent, followed by anti-C and anti-K antibodies. A higher number of transfusions and the HbSβ^0^ genotype were both persistent risk factors, while older age at first transfusion appeared protective. Extended antigen matching dramatically reduced prevalence, though approximately 9% of individuals remained affected. **Conclusions**: Alloimmunization continues to challenge transfusion management in adults with SCD. Broader implementation of extended antigen matching and genotype-informed transfusion strategies may help mitigate this risk.

## 1. Introduction

Sickle cell disease [SCD] is a hereditary hematological disorder caused by a missense mutation in the HBB gene, which encodes the β-globin subunit of hemoglobin. It is believed that SCD affects about eight million people worldwide. Sickle cell disease poses significant public health issues due to its association with complex healthcare needs, reduced life expectancy, enduring impairments, and the considerable costs of medical treatment [1,2]. Sickle cell disease is considered the most prevalent monogenic condition, characterized by a complex and detrimental cycle. This cycle emerges through various interconnected mechanisms shaped by genetic factors and the polymerization of HbS. Changes in blood flow properties, more adhesive interactions that cause vaso-occlusion, endothelial damage from hemolysis, and the start of sterile inflammatory responses are all important factors that cause this condition [3,4].

The two main signs of SCD are chronic hemolytic anemia and episodes of acute painful crisis. Nonetheless, discrepancies between genotype and phenotype lead to clinical heterogeneity, which illustrates the complexity of the underlying pathophysiology of SCD [5,6]. Patients with SCD exhibit a broad spectrum of clinical manifestations, including acute crises such as vaso-occlusive crises, acute chest syndrome, and hemolytic episodes, as well as acute and chronic end-organ damage, including stroke, acute kidney impairment, and avascular necrosis [7,8]. The diverse clinical manifestations and complex pathophysiology of SCD present significant challenges in clinical management. 

There are many treatment options available that can help prevent complications, improve quality of life, extend survival, and, in some cases, even cure the disease [3,9]. These interventions encompass non-curative methods, such as blood transfusions and disease-modifying therapies, such as hydroxyurea, as well as curative strategies, such as stem cell transplantation and gene therapy [9,10]. Among the various therapeutic options available, red blood cell (RBC) transfusion is a critical intervention for the management of SCD. This treatment is effective in preventing or mitigating numerous short-term and long-term complications, irrespective of the patient’s hemoglobin levels. Specifically, individuals with severe anemia derive significant benefits from simple blood transfusions, while patients experiencing acute complications, such as acute chest syndrome or stroke, may require exchange transfusions, even if their baseline hemoglobin levels are relatively elevated [11,12].

For individuals with SCD, getting RBC transfusions is very important. However, the need for frequent transfusions to treat this condition is very expensive, time-consuming, and comes with a lot of complications. These complications include iron overload, increased risk of infection, and alloimmunization [12,13]. Alloimmunization occurs with greater frequency in patients diagnosed with SCD compared to other populations, resulting in elevated morbidity and mortality rates. It is estimated that approximately 30% to 50% of transfused patients with SCD will develop red blood cell (RBC) alloantibodies over their lifetime, which complicates the process of identifying compatible RBCs. Notably, there exists a weak correlation between the number of transfusions and the development of alloimmunization; certain patients may receive numerous RBC transfusions without generating antibodies, while others may form multiple antibodies after only a few transfusions. Additionally, most of these alloantibodies may diminish or fall below detectable levels using traditional blood banking methodologies over time. Furthermore, Rh alloimmunization may arise despite the administration of serologically Rh-matched RBCs. On the other hand, the management of complications associated with RBC alloantibodies, including delayed hemolytic transfusion reactions (DHTRs) and the subsequent identification of compatible RBCs for future transfusions, poses significant challenges for hematologists and practitioners in transfusion medicine. These challenges persist throughout the transition period and remain pertinent in the context of advancing toward curative therapies such as hematopoietic stem cell transplantation and gene therapy [13,14].

This systematic review and meta-analysis aim to examine the global prevalence of alloimmunization among adults with SCD and identify predictors of its development, to mitigate the risk of alloimmunization and address transfusion-related challenges effectively.

## 2. Materials and Methods

### 2.1. Search Strategy and Study Selection

This systematic review and meta-analysis were performed using the PRISMA 2020 standards for the reporting of systematic reviews and meta-analyses (Table S1) [15]. Before we started any portion of the systematic review and meta-analysis [16], we developed our study protocol and registered it with PROSPERO [CRD420251167042].

A comprehensive search was performed across several major electronic databases—PubMed, Embase, Web of Science, Scopus, and the Cochrane Library—covering all entries from their inception through 8 November 2024. The literature search was subsequently updated using database sources through 16 April 2026. The search method utilized a combination of Medical Subject Headings [MeSH] phrases and free-text keywords related to the core themes of sickle cell disease, blood transfusion, and alloimmunization. The search string included terms such as “sickle cell disease,” “sickle cell anemia,” “hemoglobin SS,” “blood transfusion,” “red blood cell transfusion,” “alloimmunization,” “alloantibodies,” “red cell antibodies,” and related synonyms, combined using appropriate Boolean operators [AND, OR] to maximize sensitivity while maintaining specificity (Table S2). The reference lists of all included studies and relevant review articles were manually screened to identify additional eligible studies that the electronic search may have missed. During the initial screening phase, no language restrictions were applied. However, in the later stages, we included only those studies for which full-text articles were available in English.

Two reviewers first screened all identified records, first by title and abstract, and subsequently by full-text review, to determine eligibility for inclusion. Any disagreements between the two reviewers regarding study selection were resolved through discussion and consensus, with involvement of a third senior reviewer when necessary. Studies were selected if they fulfilled the predetermined eligibility requirements.

### 2.2. Eligibility Criteria

Studies were eligible for inclusion if they investigated the prevalence of alloimmunization in adults aged 18 years or older with confirmed sickle cell disease who received red blood cell transfusions. All original research study designs were considered eligible, including prospective cohorts, retrospective cohorts, cross-sectional studies, and randomized controlled trials [RCTs]. Studies were required to report sufficient data to calculate alloimmunization prevalence, including the number of alloimmunized patients and the total number of patients screened or transfused. Studies were excluded if they focused on pediatric populations only without adult data reported or inseparable in the study outcomes [defined as patients under 18 years of age], did not report alloimmunization outcomes as a primary or secondary endpoint, lacked sufficient data for prevalence calculation, or consisted of case reports, case series with fewer than ten cases, and any editorials, commentaries, or reviews without primary data were not included. Studies conducted in non-human subjects or in vitro investigations were also excluded. When multiple publications reported on overlapping patient populations from the same institution or cohort, the most recent or most comprehensive publication was included to ensure that no data were duplicated.

### 2.3. Data Extraction and Management

Data extraction was carried out independently by two reviewers. The information collected included key study details such as the lead author, year of publication, country and region, study design, study timeframe, and length of follow-up, participant demographics [sample size, mean or median age, age range, gender distribution, and sickle cell genotype distribution including HbSS, HbSC, and beta-thalassemia variants], transfusion characteristics [transfusion type, frequency, mean or median number of transfusion units or episodes, proportion enrolled in chronic transfusion programs, transfusion indications, red blood cell matching protocols, specific antigens matched, and use of leukoreduction], alloimmunization outcomes [number and proportion of alloimmunized patients, total number of alloantibodies detected, mean antibodies per alloimmunized patient, proportion with multiple alloantibodies, antibody detection methods, and screening frequency], antibody specificity data [most common alloantibodies identified, specific frequencies for Rh system and Kell system antibodies], complications [presence of autoantibodies, direct antiglobulin test positivity, delayed and acute hemolytic transfusion reactions, transfusion difficulty, and other reported complications], and risk factor analyses [effect estimates for age, sex, transfusion burden, genotype, matching protocol impact, and other investigated risk factors with corresponding statistical measures and *p*-values].

Discrepancies in data extraction between the two authors were identified and resolved through discussion and re-evaluation and investigation of the source documents, with arbitration by a third senior reviewer when consensus could not be reached.

### 2.4. Risk of Bias Assessment

The methodological quality and risk of bias of included observational studies were assessed by two authors using the Newcastle–Ottawa Scale [NOS] [17]. The NOS evaluates three domains: selection of study groups [zero to four stars], comparability of groups on the basis of design or analysis [zero to two stars], and ascertainment of the outcome of interest [zero to three stars], resulting in a total score ranging from zero to nine stars. Studies scoring between seven to nine stars were classified as good quality, four to six stars as fair quality, and zero to three stars as poor quality. Each study was evaluated for specific quality indicators including representativeness of the exposed cohort or sample, selection of the non-exposed cohort or comparison group when applicable, ascertainment of exposure through secure records or structured interviews, demonstration that the outcome of interest was not present at the start of the study, adequate control for confounding factors in study design or analysis, proper assessment of outcomes through blind assessment or record linkage, sufficient length of follow-up for outcomes to occur, and adequacy of follow-up with accounting for losses to follow-up.

Individual quality assessments were tabulated with detailed justification for scoring decisions, and an overall risk of bias summary was generated across all included studies. Studies were categorized as having low, moderate, or high overall risk of bias based on the aggregate evaluation of methodological strengths and limitations.

### 2.5. Statistical Analysis and Meta-Analysis

The primary outcome measure was the prevalence of alloimmunization, defined as the proportion of transfused patients who developed one or more red blood cell alloantibodies. Individual study prevalences were calculated as the number of alloimmunized patients divided by the total number of patients screened, with 95% confidence intervals [CI] computed using the Wilson Score method with continuity correction to account for the binomial distribution of proportions and to provide accurate interval estimates, especially for studies with extreme proportions or small sample sizes. To stabilize variance and normalize the distribution of proportions for meta-analysis, effect sizes were transformed using the Freeman–Tukey double arcsine transformation, which is specifically recommended for meta-analysis of prevalence data and provides superior properties compared to untransformed proportions or simple arcsine transformations.

Pooled prevalence estimates were calculated using the DerSimonian–Laird random-effects meta-analysis model, which was selected a priori based on the anticipated clinical and methodological heterogeneity across included studies arising from differences in populations, geographic regions, transfusion protocols, matching strategies, antibody detection methods, and study designs. The random-effects model assumes that the true effect size varies across studies due to real differences in study populations and methods and provides a more conservative estimate of the pooled effect with wider 95% CIs compared to fixed-effects models.

Individual study weights were calculated as the inverse of the total variance, which includes both within-study variance and between-study variance [tau-squared]. The pooled estimate was back-transformed from the Freeman–Tukey scale to the original prevalence scale for presentation and interpretation. All prevalence estimates and 95% CIs were expressed as percentages.

### 2.6. Assessment of Heterogeneity

Statistical heterogeneity was assessed using the I^2^ statistic, τ^2^, and Cochran’s Q test. Cochran’s Q was calculated as the weighted sum of squared deviations of individual study effects from the pooled estimate and evaluated using a chi-square test with k^−1^ degrees of freedom, where k is the number of included studies. A *p*-value < 0.10 for Cochran’s Q was considered indicative of statistically significant heterogeneity due to the low power of this test when few studies are included. The I^2^ statistic quantified the proportion of total variation due to heterogeneity rather than chance, with values interpreted as follows: 0–40% (may not be significant), 30–60% (moderate), 50–90% (substantial), and 75–100% (considerable heterogeneity). Tau-squared (τ^2^) was estimated using the DerSimonian-Laird method and represents the between-study variance, providing an absolute measure of heterogeneity. Potential sources and implications of heterogeneity were explored via subgroup analyses and meta-regression.

#### Subgroup Analyses and Meta-Regression Modeling

To investigate the possible underlying sources of heterogeneity and investigate effect modification by key study-level characteristics, we conducted subgroup analyses stratifying studies by geographic region [North America, South America, Middle East/Europe], study design [retrospective cohort, prospective cohort, cross-sectional], time period of publication [studies published before 2010 versus 2010 and later], quality score category [NOS score ≥ six stars versus < six stars], red blood cell matching protocol [standard ABO/RhD only, partial extended matching, full extended phenotype matching], and presence or absence of leukoreduction. For each subgroup, separate pooled prevalence estimates with 95% CIs were calculated using the random-effects model. Between-subgroup heterogeneity was assessed using the Q-between statistic with a chi-square test, where a significant *p*-value indicates that the prevalence estimates differ significantly between subgroups and that the subgroup variable explains a portion of the overall heterogeneity.

The association between alloimmunization prevalence estimates and continuous study-level factors was examined using meta-regression modeling. Covariates investigated included publication year, mean or median patient age, percentage of female participants, sample size, mean or median number of transfusion units, and proportion of patients with HbSS genotype when sufficient data were available.

Meta-regression models were fit using the logit-transformed prevalence estimates with standard errors adjusted for between-study heterogeneity. The strength and direction of associations were quantified using regression coefficients with corresponding 95% CIs and *p*-values, and Pearson correlation coefficients were calculated to describe the magnitude of linear relationships. We made bubble plots to show the connections between covariates and prevalence estimates. The diameters of the circles were based on the study weights [inverse variance]. The threshold for statistical significance in meta-regression was established at a *p*-value of less than 0.05. Because of the limited number of studies included, multivariable meta-regression models were not used to avoid overfitting. There were few studies that met the criteria, and there was a lot of variation between assays, matching protocols, and study designs.

### 2.7. Publication Bias Assessment

Small-study effects and publication bias were assessed using statistical and visual methods. A funnel plot was created with study prevalence estimates on the x-axis and standard errors on the y-axis, with 95% CI limits forming a funnel and a vertical line showing the pooled estimate. Asymmetry around the pooled estimate was evaluated as an indication of heterogeneity, small-study effects, or publication bias.

Two statistical techniques used to assess for funnel plot asymmetry were Egger’s linear regression test and Begg’s rank correlation test. Egger’s test performs a weighted linear regression of standardized effect estimates in relation to precision [the inverse of standard error]. It checks to see if the null hypothesis is true, which says that the intercept is zero. If the intercept is not zero [*p*-value < 0.10], it indicates the presence of asymmetry potentially attributable to publication bias or small-study effects. Begg’s test calculates Kendall’s tau-b rank correlation coefficient between standardized effect estimates and their variances. If the association is substantial [*p*-value < 0.05], it suggests publication bias.

#### Software and Statistical Considerations

All statistical analyses were performed using RStudio 2024.12.0 software with R version 4.4.2, with utilization of the proper meta-analysis and statistical packages. Forest plots, funnel plots, and meta-regression scatter plots were generated to visualize study-level and pooled estimates along with measures of uncertainty and heterogeneity. A two-tailed test was used for all statistical analyses, and unless otherwise stated, a *p*-value of less than 0.05 was considered statistically significant.

## 3. Results

### 3.1. Study Selection, Characteristics and Risk of Bias Assessment

**Figure 1:**
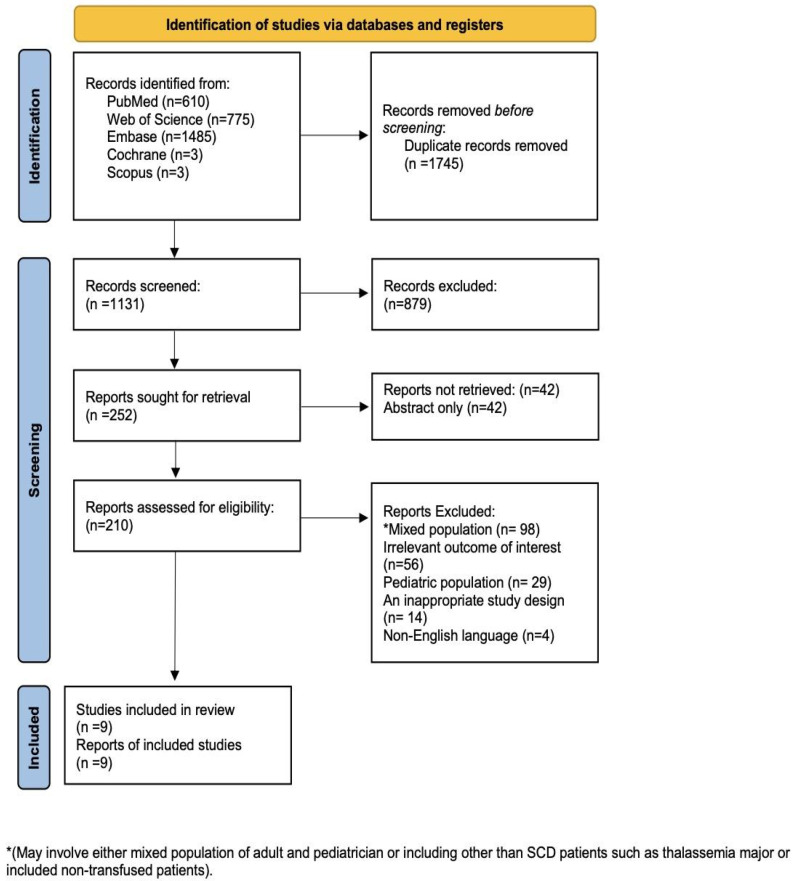
PRISMA flow diagram.

The literature search identified 2876 records up to 16 April 2026. After removing 1745 duplicates, 1131 unique citations underwent title and abstract screening. After excluding 879 records that did not meet the eligibility criteria, we assessed 210 full-text articles. Of these, 201 were excluded for various reasons: 29 focused on pediatric populations, 56 did not report alloimmunization outcomes, and 14 had inappropriate study designs. As a result, nine studies met the inclusion criteria and were included in our analysis [18,19,20,21,22,23,24,25,26] (Figure 1).

The baseline characteristics of included studies are presented in Table 1. Studies were published between 1978 and 2026, spanning 48 years. Geographically, four studies were conducted in North America [United States], three in South America [Brazil], and two in the Middle East and Turkey [Saudi Arabia and Turkey]. Study designs consisted of seven retrospective cohorts, two cross-sectional studies, and one genome-wide association study with a retrospective design. The pooled sample included a total of 1711 adult patients with sickle cell disease receiving red blood cell transfusions. Mean or median ages ranged from 29.8 to 35.7 years. Quality assessment using the NOS scale is summarized in Supplementary Table S3.

### 3.2. Patient Demographics and Genotype Distribution

Demographic characteristics are summarized in Table 2. Gender distribution showed 44.6% male and 55.4% female participants overall, with individual studies ranging from 49.4% to 65.3% female. Sickle cell genotype distribution varied, with homozygous HbSS disease predominating in most cohorts [73–100% where reported]. HbSC disease was present in four studies [2.8–14%], and beta-thalassemia variants represented 5–15.6% in studies reporting these genotypes. North American studies enrolled mostly African American patients [99–99.2% African ancestry], South American studies included patients of African or mixed ancestry [>82% African], and Middle Eastern studies enrolled Saudi Arabian and Turkish patients of Arab and Mediterranean ancestry, respectively.

### 3.3. Transfusion Characteristics and Matching Protocols

Transfusion characteristics are detailed in Table 3. Five studies included both simple and exchange transfusions; four reported mixed types. Transfusion frequency varied, with six studies including both chronic regular and episodic transfusions. Mean or median transfusion burden ranged from 4.0 to 23.5 units, where reported. Chronic transfusion program enrollment ranged from 3.8% to 100% across studies. Indications included acute chest syndrome, stroke prevention, chronic pain, severe anemia, vaso-occlusive crises, and pregnancy. Red blood cell matching protocols evolved substantially: one 1978 study used only ABO/RhD matching, four utilized partial extended matching [typically C, E, K], three used detailed extended phenotype matching [more than four, typically 8–9 antigens], and one Saudi study transitioned from standard to partial extended matching in 2013. Leukoreduction was documented in four studies, absent in one historical study, and unreported in four studies.

### 3.4. Alloimmunization Prevalence and Heterogeneity

**Figure 2:**
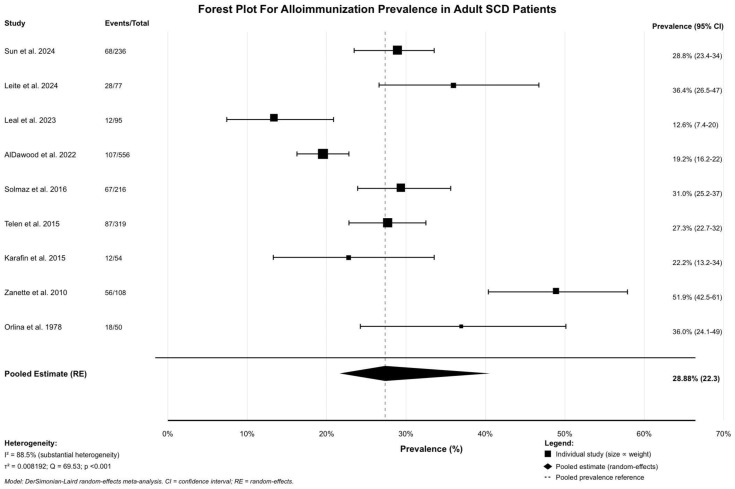
Forest plot for alloimmunization prevalence [18,19,20,21,22,23,24,25,26].

We performed the Egger’s linear regression test and the Begg’s rank correlation test to see if the funnel plot was symmetrical. Table 4 displays the primary outcome results. Out of 1711 patients, 455 produced alloantibodies, leading to a crude prevalence rate of 26.59%. The prevalence of each study ranged from 12.6% to 51.9%. The random-effects meta-analysis indicated that the overall prevalence was 28.88% [95% CI: 22.37–35.38%], as shown in Figure 2. This means that around one out of every three adults who get a transfusion gets alloimmunization. There were a lot of asymmetries, with I^2^ = 88.5%. This suggests that 88.5% of the difference was because of actual differences and not sampling error. Cochran’s Q statistic was 69.53 [df = 8, *p*-value < 0.001], which demonstrated that there was a lot of difference among studies. The difference between studies [τ^2^] was 0.008192. Various laboratory methods were used to detect alloantibodies, including gel card technology, standard serology, genotyping, and solid-phase assays; in addition, some studies identified alloantibodies through chart review. Studies examining total antibodies identified a range between 36 and 58, with each alloimmunized patient exhibiting 2.00 to 2.07 antibodies. In each group, 5 to 36 patients had more than one alloantibody. Six trials with between 14 and 60 patients each identified an autoantibody.

### 3.5. Subgroup Analysis by Geographic Region

**Figure 3:**
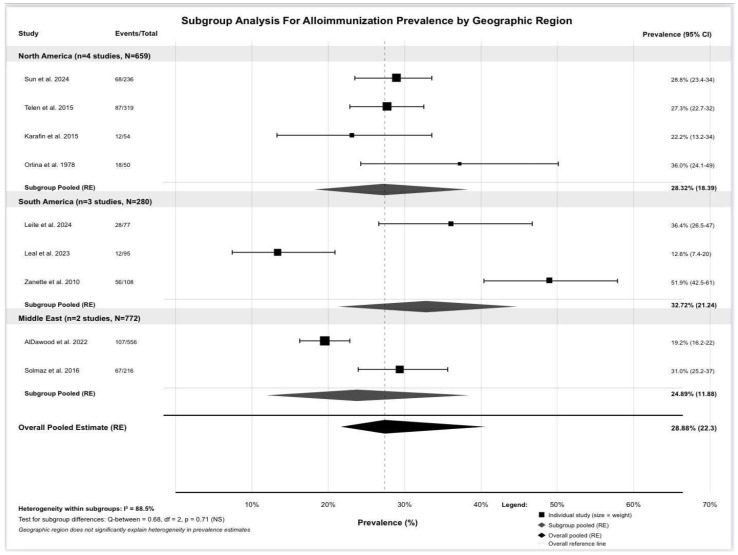
Forest plot for subgrouping by geographic region [18,19,20,21,22,23,24,25,26].

Our subgroup analysis by geographic region is demonstrated in Figure 3. North American studies [four studies, 659 patients] yielded a pooled prevalence of 28.32% [95% CI: 18.39–38.25%]. South American studies [three studies, 280 patients] demonstrated 32.72% [95% CI: 21.24–44.20%]. Middle Eastern and Turkish studies [22] [two, 772 patients] showed 24.89% [95% CI: 11.88–37.91%]. The test for subgroup differences resulted in Q-between = 0.68 [df = 2, *p*-value = 0.71], indicating no significant differences between regions. This suggests that geographic region was not found to explain observed heterogeneity, and other factors such as transfusion protocols, matching strategies, and detection methods may be more important contributors. Significant within-subgroup heterogeneity persisted across all regions.

### 3.6. Alloantibody Specificity and Complications

Antibody specificities and complications are summarized in Table 5. Rh system antibodies were most frequent, with anti-E documented in six studies [7–46% of alloimmunized patients were quantified, nine to 37 cases]. Anti-C was the second-most common, identified in five studies [3–35.6%, three to 15 cases]. Kell system antibodies, particularly anti-K, were documented in four studies [four to 33 cases]. Other antibodies included anti-D [three cases], anti-S [29.9% in one study], anti-Leᵃ [10 cases in a historical study], and antibodies to low-prevalence antigens [five cases]. Autoantibody formation occurred in six studies [14–60 cases, 6.5–21.3% prevalence]. Direct antiglobulin test positivity was reported in four studies [16–60 patients]. Delayed hemolytic transfusion reactions were documented in two studies [five cases reported]. Transfusion difficulty was noted in two studies, with one reporting 9.3% developed new antibodies despite extended matching. Other complications included chronic pain associations [two studies], avascular necrosis [one study], and differential survival outcomes [hazard ratio [HR] 1.92 in one study; no association in another, *p*-value = 0.25].

### 3.7. Risk Factors Analysis and Meta-Regression

**Figure 4:**
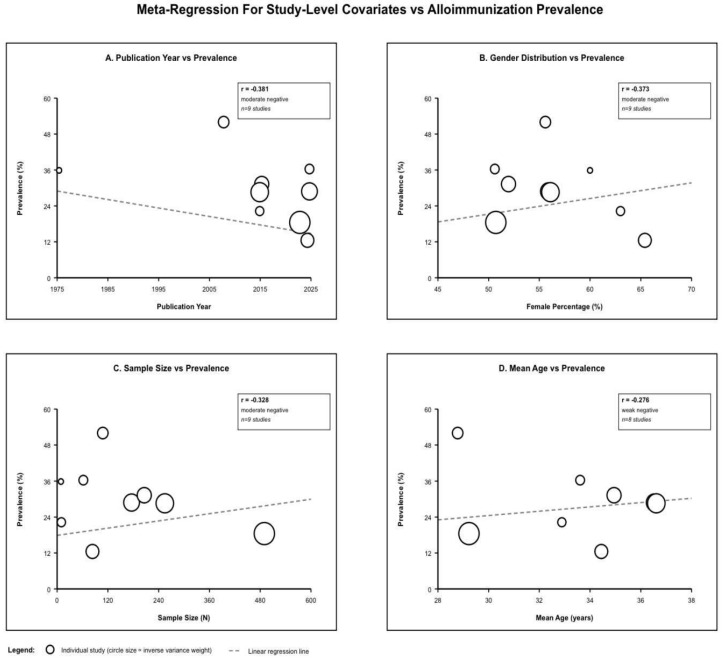
Meta-regression plot.

Risk factor analyses are presented in Table 6 and visualized in Figure 4. Age showed inconsistent associations: one Saudi cohort found an odds ratio [OR] 1.08 for older age [*p*-value < 0.05] [21], one Brazilian study identified younger age as a risk [*p*-value = 0.04], while three studies found no association. Meta-regression demonstrated a weak negative correlation between mean age and prevalence [r = −0.276, non-significant]. Female sex was identified as a risk factor in two studies [OR 2.19 in one; *p*-value= 0.03 in another], while three found no association [*p*-value= 0.10, 0.15, and 0.90]. Meta-regression demonstrated a moderate negative correlation between female percentage and prevalence [r = −0.373], possibly reflecting confounding bias. Transfusion burden was the most consistent risk factor: three studies found significant associations [20.9 vs. 9.8 units, *p*-value = 0.001; 15.7 vs. 7.8 units; univariate *p*-value < 0.001 but multivariable *p*-value = 0.74 in one large cohort]. Genotype demonstrated HbSβ^0^ as a risk factor in two cohorts [*p*-value = 0.004, *p*-value = 0.001], while one found no association [*p*-value = 0.34]. Extended matching reduced prevalence from 47% to 12.6% in one Brazilian study [73% relative reduction], though 9.3% still developed antibodies despite matching in another study. Other factors were DAT positivity [OR 10.23], older age at first transfusion [which is protective but inconsistently reported across studies], the presence of autoantibodies, chromosome 12 genetic loci, and RH variant alleles.

Meta-regression analysis of publication year demonstrated a moderate negative association [r = −0.381], suggesting a temporal trend towards reduced incidence in more recent research. Sample size exhibited a moderate negative association [r = −0.328], indicating that larger studies yielded lower values. Overall heterogeneity remained substantial [I^2^ = 88.5%, τ^2^ = 0.008192, Q = 69.53], indicating that the evaluated factors explained negligible variance. Using statistical techniques to look for funnel plot asymmetry gave notable results.

Egger’s linear regression test resulted in an intercept of 38.733 [95% CI: 20.43–57.04], t-statistic 5.004 [df = 7], with a statistically significant *p*-value of 0.010. Begg’s rank correlation test resulted in Kendall’s tau of 0.3333 [z-statistic 1.251], with a *p*-value of 0.0275, indicating significant positive rank correlation between effect size and variance, with 24 concordant pairs versus 12 discordant pairs among 36 possible comparisons.

### 3.8. Publication Bias Assessment

**Figure 5:**
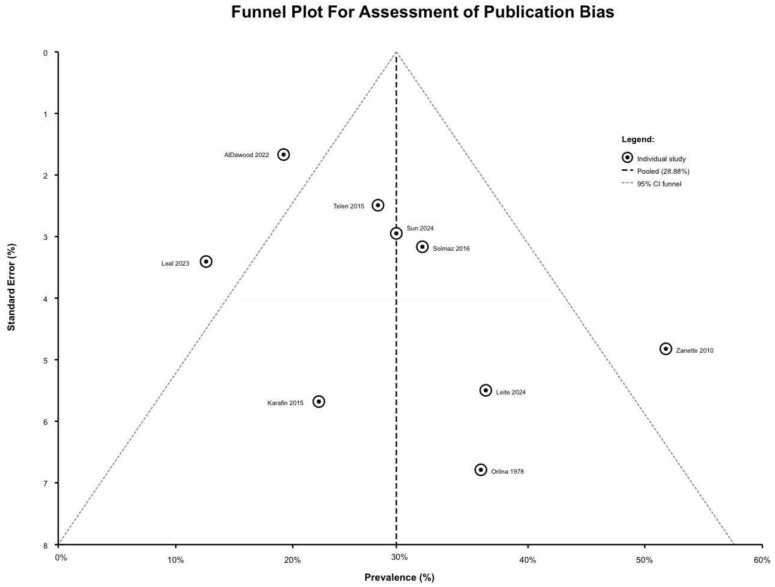
Funnel plot of asymmetry for publication bias assessment [18,19,20,21,22,23,24,25,26].

Publication bias assessment is presented through a visual inspection funnel plot in Figure 5. Visual inspection showed a relatively symmetric distribution around the pooled estimate of 28.88%, with studies distributed across standard errors from 1.67% to 6.79%. Four studies reported prevalence above the pooled estimate [31.0–51.9%], five below [12.6–28.8%], suggesting a reasonable balance. However, statistical tests demonstrated potential evidence of asymmetry. Despite significant statistical tests, multiple factors suggest that detected asymmetry reflects clinical heterogeneity rather than a true publication bias effect, including visual symmetry without obvious gaps, significant documented heterogeneity [I^2^ = 88.5%] from identifiable sources, no regional differences in subgrouping [Q = 0.68, *p*-value= 0.71], and a wide prevalence range [12.6–51.9%, spanning 39.3 percentage points] reflecting clinical differences and variability.

The studies were almost evenly split between those above [four studies, 44.4%] and those below [five studies, 55.6%] the pooled estimate. The overall risk of publication bias is deemed moderate, with significant caveats, leading to the conclusion that statistical asymmetry likely reflects clinical heterogeneity among diverse populations and methodologies rather than structural statistical bias.

## 4. Discussion

The transfusion of blood components represents a vital component of contemporary medical practice in the treatment and care of patients. Red blood cell alloimmunization significantly complicates transfusion therapy, especially for patients reliant on frequent transfusions, including those with hemoglobinopathies such as thalassemia and SCD. These patients are at a heightened risk of developing alloimmunization, which may result in significant complications and challenges in identifying compatible blood sources [27,28]. This systematic review and meta-analysis compiles contemporary evidence regarding the prevalence of alloimmunization and the factors that facilitate its occurrence in individuals with sickle cell disease [SCD]. It utilizes research published from 1978 to 2026 and includes findings from 1711 adult patients with SCD who underwent red blood cell transfusions.

Alloimmunization rates exhibit considerable variability among individuals undergoing blood transfusions. Reports indicate that the prevalence of alloimmunization ranges from 4% to 50% in patients with thalassemia, from 1.9% to 13% in oncology and hematology patients, and from 1.27% to 13.1% in renal patients [28]. This review determined that approximately one-third of individuals with SCD who receive transfusions display alloimmunization, with prevalence rates varying between 12.6% and 51.9% [27,28]. This figure is nearly double that reported for other chronic conditions that necessitate blood transfusions and slightly exceeds the frequency observed in thalassemia patients [27,29]. In our review, we observed that the rate of alloimmunization is approximately double that in pediatric patients with sickle cell disease. This discrepancy can be partially attributed to the higher frequency of transfusions experienced by the adult patient population compared to their pediatric counterparts [30,31]. Specifically, it is estimated that one in every 3.5 transfused adults develops alloimmunization, with an average of two antibodies identified per alloimmunized patient. Contrary to previous findings, the notable geographical variations among the pooled study populations do not appear to significantly affect the prevalence of alloimmunization and do not adequately account for the observed heterogeneity. The influence of ethnicity on the risk of alloimmunization remains challenging to determine due to the predominance of participants of African ancestry in the existing literature. There is a limited representation of individuals from the Mediterranean region and other ancestral backgrounds, where distinct haplotypes may be present [32].

Sensitization to Rh and K antigens is prevalent as these erythrocyte antigens exhibit significant immunogenicity. Consequently, it is recommended that RBCs be provided with phenotype matching for ABO blood groups and the commonly encountered Rh antigens [C/c, E/e], along with K antigens, as a strategy to mitigate alloantibody formation [33,34]. In our study, anti-E was identified as the most prevalent antibody, followed by anti-C and antibodies from the Kell system, anti-D and anti-S. It is noteworthy that antibodies such as anti-E, anti-C, and anti-S are associated not only with increased incidences of delayed hemolytic transfusion reactions and transfusion challenges but also with additional complications such as chronic pain and avascular necrosis. In alignment with these recommendations, our findings indicate that extended red cell antigen typing significantly decreases the rate of alloimmunization. However, it is still important to remember that up to 10% of patients still have alloimmunization even after extended phenotyping has been put in place. Recent studies indicate a temporal trend towards a declining prevalence of alloimmunization, potentially attributable to increased awareness and compliance with established management guidelines.

New evidence shows that the risk of alloimmunization after a red blood cell transfusion is affected by many things, such as the donor’s characteristics, the components of the blood, and the host’s characteristics. Important host factors are blood-group antigen negativity, HLA type, TRIM21 gene polymorphism, recipient inflammation, and how often transfusions are given [32,35]. Our study demonstrated that transfusion burden is the most reliable risk factor for the onset of alloimmunization. Moreover, the HbSβ^0^ genotype was recognized as a risk factor for alloimmunization in comparison to other genotypes, including HbSS. Significantly, a higher age at the initial transfusion correlated with a diminished risk of alloimmunization. This finding contradicts prior studies that suggested a higher incidence of alloimmunization typically occurs post-five years of age [36]. Beginning transfusions at a young age, less than five years old, may foster immune tolerance due to the underdeveloped state of the immune system, thus providing a safeguard against alloimmunization. Additionally, various immune alterations linked to SCD exhibit parallels to immunosenescence seen in the elderly, such as compromised phagocytosis and a diminished count of naive T cells [32,37]. In other words, the diminished risk of alloimmunization observed at the extremes of age may be attributed to the underdevelopment of the immune system in early life, as well as to immune system compromise that occurs with advancing age. In addition, we conducted an analysis of several additional factors, including gender, the presence of autoantibodies, genetic loci on chromosome 12, and RH variant alleles. However, the results for these factors did not reach statistical significance.

This study clarifies the worldwide prevalence of alloimmunization in patients with SCD, which continues to be widespread in different geographical areas. It emphasizes the critical role of transfusion burden in the onset of alloimmunization, in conjunction with factors such as particular sickle cell genotypes and the age at which the initial transfusion occurs. The study also shows that extended phenotyping for ABO and [C, E or C/c, E/e] and K antigens can significantly lower the rate of alloimmunization. Conversely, demographic factors, including gender and age, were not identified as reliable indicators of alloimmunization. The study, however, recognizes several limitations, notably its concentration on adult populations while omitting pediatric age groups (which were found to have lower prevalence rates of alloimmunization as compared to adults). Additionally, the frequency and nature of transfusions, as well as functional or anatomical splenectomy and the concomitant risk of alloimmunization, were not central elements of the examined literature. These results indicate that employing strategies to postpone blood transfusions or reduce their frequency, such as commencing disease-modifying therapies at an earlier age and following established guidelines for transfusion thresholds, may substantially impact the rate of alloimmunization. Likewise, rigorous adherence to guidelines regarding extended phenotyping may persist in reducing the risk of alloimmunization [38,39]. Nonetheless, the incidence of alloimmunization despite the application of these measures signifies the necessity to examine additional identified antibodies, such as anti-S, found in approximately one-third of patients, as well as to assess other donor attributes and transfused components. Moreover, prior research has demonstrated that certain individuals with SCD developed antibodies despite having lower transfusion rates relative to other SCD patients and those with different chronic transfusion needs, such as individuals with thalassemia [23,40]. Consequently, further research is imperative to examine the rate of alloimmunization, taking into account variables such as vaccination status, the nature of the transfusion [simple or exchange], and the timing and rationale for transfusion, whether it is administered acutely or as part of a chronic treatment regimen. These factors may elucidate the persistent inflammatory condition. Notably, studies implementing truly extended red blood cell matching that includes Rh (C, c, E, e), Kell, Duffy, and Kidd antigens demonstrate substantially lower alloimmunization prevalence, supporting the use of comprehensive phenotypic matching over limited or partial matching strategies in chronically transfused populations.

The strengths of this study are that it is registered with PROSPERO with set methods, strictly follows the PRISMA 2020 guidelines, and uses a thorough multi-database search strategy. The review is distinctive in its focus on adult populations, covering a broad temporal range [1978–2024], and offers a comprehensive extraction of antibody specificities [Rh/Kell systems] with evident clinical implications. Furthermore, the origins of heterogeneity and potential predictors were examined through subgroup and meta-regression analysis.

Nonetheless, several limitations of this study should be acknowledged. First, the number of studies meeting the inclusion criteria was relatively limited, and considerable heterogeneity existed across assay methodologies, matching protocols, and study designs. Most of the included studies were retrospective, which may introduce selection and reporting biases. Furthermore, geographic representation across the included studies was limited, and the meta-regression analysis may have lacked sufficient statistical power to fully account for heterogeneity. An individual patient data meta-analysis was not feasible due to restricted access to raw datasets, variations in reporting formats, and limitations imposed by study authors, which constrained a more detailed evaluation of potential risk factors. In addition, several studies reported combined pediatric and adult populations without providing separate subgroup analyses. As a result, these studies could not be fully incorporated into subgroup analyses, contributing to a lower retrieval rate of eligible data and limiting the ability to assess age-specific differences. The funnel plot demonstrated asymmetry, with smaller studies reporting lower prevalence appearing underrepresented, and this observation was supported by a statistically significant Egger’s regression test, suggesting publication bias. Consequently, the pooled prevalence estimates may be overestimated and should therefore be interpreted with caution. Finally, incomplete reporting of important clinical variables, including transfusion protocols, splenectomy status, and standardized outcome definitions, may have influenced the pooled estimates and reduced the generalizability of the findings.

## 5. Conclusions

In conclusion, blood transfusion is a critical component in the management of patients with SCD. However, alloimmunization presents a significant challenge within blood transfusion practices, particularly for patients who require immediate transfusion. Despite considerable advancements in the field and the implementation of more stringent, specific guidelines for transfusion, especially regarding extended antigen matching, individuals continue to develop alloantibodies. In this context, the International Collaboration for Transfusion Medicine Guidelines recommends that red blood cell units for patients with SCD should be matched for Rh antigens (D, C, E, c, e) and K antigens to reduce the risk of alloimmunization [41]. This issue may be addressed by applying comprehensive serological and genomic techniques when feasible. Moreover, strategies to reduce transfusion burden and delay the initiation of transfusion in a patient’s life are of utmost importance. These strategies may involve modifiable agents such as hydroxyurea, as well as curative options, including stem cell transplantation and gene therapy, which are fundamental for minimizing the risk of alloimmunization. Further research is warranted to investigate the risk of alloimmunization among individuals with long-term hydroxyurea therapy, as well as those with persistent splenomegaly, in contrast to individuals who have undergone functional or anatomical splenectomy. Additionally, the indications for transfusion and the subsequent development of alloimmunization merit further examination, as they remain inadequately addressed in the current literature. The influence of ethnicity on alloimmunization risk should be considered in future research, even in the absence of geographical variation, particularly given the predominance of participants of African ancestry and the underrepresentation of individuals from other ancestral backgrounds with distinct haplotypes. Finally, it is imperative to enhance transfusion protocols and identify novel risk factors to facilitate the prevention and reduction of future red blood cell alloimmunization. This focus is particularly crucial at this time, given the absence of effective treatments for patients who are sensitized.

## Figures and Tables

**Table 1:** Included studies characteristics and baseline demographics of included studies.

Study	Country	Study Design	Study Period	Follow-Up Duration	Total Sample Size [N]	Mean/Median Age [Years]	Age Range [Years]	Quality Rating
Sun et al., 2024 [18]	USA	GWAS/Retrospective Cohort	2002–2005 [enrollment]	NR	236	35.7 [mean]	≥18	High
Leite et al., 2024 [19]	Brazil	Cross-sectional	NR	NR	77	33.0 [mean]	NR	High
Leal et al., 2023 [20]	Brazil	Retrospective Cohort	20-year period	20 years	95	34 [median]	NR	High
AlDawood et al., 2022 [21]	Saudi Arabia	Retrospective Cohort	2010–2018	9 years	556	29.8 [median]	NR	High
Solmaz et al., 2016 [22]	Turkey	Retrospective Cohort	2011–2015	4 years	216	33.9 [mean]	≥18	Medium
Telen et al., 2015 [23]	USA	Retrospective Cohort	2002–2005 [enrollment]	NR	319	35.1 [mean]	≥18 [18–84]	High
Karafin et al., 2015 [24]	USA	Retrospective Cohort	2010–2013	3 years	54	32.4 [mean]	NR	High
Zanette et al., 2010 [25]	Brazil	Cross-sectional	2004–2007	4 years	108	30.1 [allo] vs. 34.3 [non-allo]	18–61	High
Orlina et al., 1978 [26]	USA	Retrospective Cohort	33 months [1973–1976]	33 months	50	NR	19–49	High

Abbreviations: GWAS, genome-wide association study; NR, not reported; allo, alloimmunized; non-allo, non-alloimmunized.

**Table 2:** Patient demographics and SCD genotype distribution.

Study	Male n [%]	Female n [%]	HbSS [%]	HbSC [%]	HbSβ-thal [%]	HbSβ^0^ [%]	HbSβ+ [%]	Race/Ethnicity	Geographic Region	Population Ancestry
Sun et al., 2024 [18]	104 [44.1]	132 [55.9]	73	14	NR	6	7	99.2% Black	North America	African
Leite et al., 2024 [19]	38 [49.4]	39 [50.6]	NR	NR	NR	NR	NR	NR	South America	NR
Leal et al., 2023 [20]	33 [34.7]	62 [65.3]	100	0	0	NR	NR	NR	South America	NR
AlDawood et al., 2022 [21]	274 [49.3]	282 [50.7]	84.4	0	15.6	NR	NR	96.2% Saudi	Middle East	Arab
Solmaz et al., 2016 [22]	104 [48.1]	112 [51.9]	73.1	0	NR	13.0	13.9	Turkish	Middle East/Europe	Mediterranean
Telen et al., 2015 [23]	140 [43.9]	179 [56.1]	77	13	NR	5	5	99% African American	North America	African
Karafin et al., 2015 [24]	20 [37.0]	34 [63.0]	NR	NR	NR	NR	NR	African American	North America	African
Zanette et al., 2010 [25]	48 [44.4]	60 [55.6]	97.2	2.8	0	NR	NR	>82% African ancestry	South America	African
Orlina et al., 1978 [26]	20 [40.0]	30 [60.0]	82	8	10	NR	NR	Black	North America	African

Abbreviations: HbSS, homozygous sickle cell disease; HbSC, sickle cell-hemoglobin C disease; HbSβ-thal, sickle cell-beta thalassemia; HbSβ^0^, sickle cell-beta zero thalassemia; HbSβ+, sickle cell-beta plus thalassemia; n, number; NR, not reported.

**Table 3:** Transfusion characteristics and RBC matching protocols.

Study	Transfusion Type	Transfusion Frequency	Mean/Median Transfusion Units or Episodes	Chronic Transfusion Program n (%)	Transfusion Indication	RBC Matching Protocol	Specific Antigens Matched	Leukoreduction
Sun et al., 2024 [18]	Mixed	Mixed	Categorized	NR	NR	Partial Extended	C, E, K	NR
Leite et al., 2024 [19]	Mixed	Mixed	NR	NR	NR	Extended	C, D, E, K, Fya/b, Jka/b, S, s	NR
Leal et al., 2023 [20]	Both	Both	Mean: 23.5 (chronic), 3.8 (episodic)	58 (61.1)	ACS, stroke prevention, pregnancy	Extended	D, C, c, E, e, K, Fya/b, Jka/b, S	Yes
AlDawood et al., 2022 [21]	Mixed	Mixed	Median: 4.0 units	NR	NR	Standard then Partial Extended	ABO/D only until 2013, then extended	NR
Solmaz et al., 2016 [22]	Both	Both	Categorized	160 (74.1)	NR	Partial Extended	ABO, Rh, Kell	Yes
Telen et al., 2015 [23]	Mixed	Both	Self-reported categories	12 (3.8)	NR	Partial Extended	C, E, K	NR
Karafin et al., 2015 [24]	Both	Regular (chronic)	Median: 31 units/3 years	54 (100)	Stroke prevention, chronic pain	Extended	C, c, E, e, K	Yes
Zanette et al., 2010 [25]	Both	Both	Mean: 14.96 (allo) vs. 10.55 (non-allo)	10 (9.3)	Severe anemia, ulcers, VOC	Partial Extended	C, c, E, e, K (since 2004)	Yes
Orlina et al., 1978 [26]	NR	Episodic	Mean: 15.7 (allo) vs. 7.8 (non-allo)	NR	Aplastic crisis, prophylactic	Standard	ABO/RhD only	No

**Table 4:** Primary outcome analysis of alloimmunization prevalence.

Study	Year	Total N	Allo n	Non-Allo n	Prev [%]	95% CI Lower	95% CI Upper	SE [%]	Variance	Logit[p]	SE Logit	FT Transform	Weight [IV]	Detection Method	Multiple Abs	Autoabs
Sun et al., 2024 [18]	2024	236	68	168	28.8	23.4	34.9	2.95	0.000869	−0.9001	0.1433	0.5676	1150.58	Chart Review	Yes, n = NR	18
Leite et al., 2024 [19]	2024	77	28	49	36.4	26.5	47.5	5.48	0.003005	−0.5521	0.2351	0.6491	332.75	Gel card [ID-DiaPanel]	Yes, n = NR	NR
Leal et al., 2023 [20]	2023	95	12	83	12.6	7.4	20.8	3.41	0.001162	−1.8991	0.3033	0.3691	860.82	Genotyping [HEA BeadChip]	NR	16
AlDawood et al., 2022 [21]	2022	556	107	449	19.2	16.2	22.7	1.67	0.000280	−1.4306	0.1074	0.4548	3577.62	ID-gel card	36	60
Solmaz et al., 2016 [22]	2016	216	67	149	31.0	25.2	37.5	3.15	0.000991	−0.7952	0.1466	0.5916	1009.49	Gel technology [ID-DiaCell]	NR	14
Telen et al., 2015 [23]	2015	319	87	232	27.3	22.7	32.4	2.49	0.000622	−0.9773	0.1254	0.5503	1608.29	Standard serology	Yes, n = NR	24
Karafin et al., 2015 [24]	2015	54	12	42	22.2	13.2	34.9	5.66	0.003201	−1.2238	0.3218	0.4969	312.43	Solid-phase, PEG/LISS tube	5	NR
Zanette et al., 2010 [25]	2010	108	56	52	51.9	42.5	61.0	4.81	0.002312	0.0734	0.1917	0.8038	432.59	NR	19	23
Orlina et al., 1978 [26]	1978	50	18	32	36.0	24.1	49.9	6.79	0.004608	−0.5635	0.2912	0.6463	217.01	Standard serology	13	NR

Abbreviations: N, total number of patients screened; Allo n, number of alloimmunized patients; Non-Allo n, number of non-alloimmunized patients; Prev, prevalence; CI, confidence interval [Wilson Score method]; SE, standard error; Logit[p], logit-transformed proportion with continuity correction; SE Logit, standard error of logit transformation; FT Transform, Freeman-Tukey double arcsine transformation; Weight [IV], inverse variance weight for fixed-effects meta-analysis; ID, indirect antiglobulin test; HEA, human erythrocyte antigen; PEG, polyethylene glycol; LISS, low ionic strength solution; Abs, alloantibodies; Abs/Pt, antibodies per alloimmunized patient; Multiple Abs, patients with multiple alloantibodies; Autoabs, autoantibodies detected; n, number; NR, not reported.

**Table 5:** Alloantibody specificity, complications, and outcome parameters.

Study	Year	Most Common Alloantibodies	Autoabs n [%]	Other Complications	Effect Sizes and Associations
Sun et al., 2024 [18]	2024	NR	18	Chronic pain assoc.	Female OR 2.19
Leite et al., 2024 [19]	2024	Anti-C [12], Anti-E [7], Other Rh [13]	NR	None	Age NS [*p* = 0.48]; Gender NS [*p* = 0.10]
Leal et al., 2023 [20]	2023	Rh Abs [7 pts], Low-prev [5 pts]	16 [16.8%]	None	Transfusion [*p* = 0.001]; Matching reduced 47%→12
AlDawood et al., 2022 [21]	2022	Anti-E [37], Anti-K [33], Anti-C [15]	60 [10.8%]	None	Age OR 1.08; DAT+ OR 10.23
Solmaz et al., 2016 [22]	2016	NR	14 [6.5%]	Allo did NOT↑mortality [*p* = 0.25]	Age NS; Gender NS [*p* = 0.90]
Telen et al., 2015 [23]	2015	Anti-E [46%], Anti-C [35.6%], Anti-S [29.9%]	24 [7.5%]	AVN, chronic pain	Survival HR 1.92; Genotype HbSβ^0^ [*p* = 0.004]
Karafin et al., 2015 [24]	2015	Anti-K [4], Anti-D [3], Anti-C [3]	NR	9.3% new Abs despite matching	Transfusion NS [*p* = 0.3];
Zanette et al., 2010 [25]	2010	Anti-E [25], Anti-K [12], Anti-C [9]	23 [21.3%]	No diff in ulcers/stroke	Age [*p* = 0.04]; Female [*p* = 0.03]; DAT+ [*p* < 0.001]
Orlina et al., 1978 [26]	1978	Anti-Leᵃ [10], Anti-E [9], Anti-Kell [5]	NR	None	Gender [F > M];

Abbreviations: Rh, Rhesus blood-group system; C, Rhesus C antigen; D, Rhesus D antigen; E, Rhesus E antigen; K, Kell antigen; S, MNS S antigen; Leᵃ, Lewis a antigen; Abs, alloantibodies; Low-prev, low-prevalence antigens; pts, patients; Autoabs, autoantibodies; DAT+, direct antiglobulin test positive; Allo, alloimmunization; AVN, avascular necrosis; assoc., association; diff, difference; OR, odds ratio; HR, hazard ratio; NS, not significant; n, number; NR, not reported; F, female; M, male; HbSβ^0^, sickle cell-beta zero thalassemia.

**Table 6:** Risk factors analysis with meta-regression.

Study (N)	Multivar Analysis	Age [Effect | *p*]	Gender [Effect | *p*]	Transfusion [Effect | *p*]	Genotype [Effect | *p*]	Matching Impact	Other Risk Factors	Region	Study Prev [95% CI]	Weight
Sun et al., 2024 (236). [18]	Yes [GWAS]	NR | NR	Female OR 2.19 | Nominal	Nominal | Nominal	HbSS/Sβ^0^ higher | 0.001	NR	Autoabs; Chr12 loci; Chronic pain	North America	28.8%	High
Leite et al., 2024 (77). [19]	No	NS | 0.48	NS | 0.10	NR | NR	NR | NR	NR	Genotype–phenotype discrepancies	South America	36.4%	Low
Leal et al., 2023 (95). [20]	No	NR | NR	NR | NR	20.9 vs. 9.8 units | 0.001	All HbSS | NA	↓47%→12.6%	RH variant alleles	South America	12.6%	Moderate
AlDawood et al., 2022 (556). [21]	Yes [Logistic]	OR 1.08 older | <0.05	NS | 0.15	Univ:<0.001; Multi:NS | 0.74	NS | 0.34	NR	DAT+ OR 10.23; Age 1st Tx protective	Middle East	19.2%	Very High
Solmaz et al., 2016 (216). [22]	No	NS | NS	NS | 0.90	NS | >0.05	NR | NR	NR	No SCD complications were risk factors	Middle East	31.0%	Moderate
Telen et al., 2015 (319). [23]	Yes [Multiple Regr.]	NS | >0.05	NS | >0.05	NS | NS	HbSβ^0^ highest | 0.004	NR	Survival HR 1.92; AVN; Pain; Autoabs	North America	27.3%	High
Karafin et al., 2015 (54). [24]	No	NR | NR	NR | NR	NS | 0.3	NR | NR	9.3% new Abs despite match	Rh variants n = 3	North America	22.2%	Low
Zanette et al., 2010 (108). [25]	No	Younger risk | 0.04	Female risk | 0.03	NS | 0.06	NR | NR	NR	DAT+ [*p* < 0.001]	South America	51.9%	Low
Orlina et al., 1978 (50). [26]	No	NR | NR	F > M | NR	15.7 vs. 7.8 units | NR	NR | NR	ABO/RhD only	Pregnancy history	North America	36.0%	Very Low
POOLED ESTIMATE (1711)	—	Meta-regr: r = −0.28	Meta-regr: r = −0.37	Varies by study	HbSβ^0^ assoc. in 2/9	Extended: 28.2%	I^2^ = 88.5%; τ^2^ = 0.008; Q = 69.53	Overall	28.88% [22.37–35.38%]	—
SUBGROUP: North America (659)	n = 4 studies	—	—	—	—	—	Q-between test: *p* < 0.05	North America	28.32% [18.39–38.25%]	—
SUBGROUP: South America (280)	n = 3 studies	—	—	—	—	—	—	South America	32.72% [21.24–44.20%]	—
SUBGROUP: Middle East (772)	n = 2 studies	—	—	—	—	—	—	Middle East	24.89% [11.88–37.91%]	—

Abbreviations: N, total sample size; Multivar, multivariable; GWAS, genome-wide association study; Regr., regression; OR, odds ratio; HR, hazard ratio; NS, not significant; NR, not reported; HbSS, homozygous sickle cell disease; HbSβ^0^, sickle cell-beta zero thalassemia; Autoabs, autoantibodies; Chr12, chromosome 12; DAT+, direct antiglobulin test positive; AVN, avascular necrosis; Tx, transfusion; Univ, univariate; Multi, multivariable; Abs, alloantibodies; F, female; M, male; I^2^, I-squared heterogeneity statistic; τ^2^, tau-squared [between-study variance]; Q, Cochran’s Q statistic; r, Pearson correlation coefficient; NA, not applicable.

## Data Availability

The data generated or analyzed in this study are contained within this published article.

## Supplementary Materials

The following supporting information can be downloaded at: https://www.mdpi.com/article/10.3390/jcm15103828/s1. Table S1: PRISMA-2020 checklist; Table S2: Search strategy used in each of the databases; Table S3: Risk of bias assessment.

## Abbreviations

The following abbreviations are used in this manuscript:

SCD	Sickle cell disease
VOC	Vaso-occlusive crisis
ACS	Acute chest syndrome
RBCs	Red blood cells
DHTRs	Delayed hemolytic transfusion reactions

## References

[1] Thomson A.M., McHugh T.A., Oron A.P., Teply C., Lonberg N., Tella V.V., Wilner L.B., Fuller K., Hagins H., Aboagye R.G. (2023). Global, regional, and national prevalence and mortality burden of sickle cell disease, 2000–2021: A systematic analysis from the Global Burden of Disease Study 2021. Lancet Haematol..

[2] Kayle M., Blewer A.L., Pan W., Rothman J.A., Polick C.S., Rivenbark J., Fisher E., Reyes C., Strouse J.J., Weeks S. (2024). Birth Prevalence of Sickle Cell Disease and County-Level Social Vulnerability—Sickle Cell Data Collection Program, 11 States, 2016–2020. MMWR. Morb. Mortal. Wkly. Rep..

[3] Alsalman M., Alsalman Z., Alkhalifa H.A., Alfaraj A.N., Alkhalifah A., Almulihi Q. (2023). Predictors of Intensive Care Admission Among Adult Patients with Sickle Cell Disease in Eastern Province of Saudi Arabia. J. Blood Med..

[4] Sundd P., Gladwin M.T., Novelli E.M. (2019). Pathophysiology of Sickle Cell Disease. Annu. Rev. Pathol. Mech. Dis..

[5] Xu J.Z., Thein S.L. (2022). Revisiting anemia in sickle cell disease and finding the balance with therapeutic approaches. Blood.

[6] Beutler E. (2001). Discrepancies between genotype and phenotype in hematology: An important frontier. Blood.

[7] Casale M., Benemei S., Gallucci C., Graziadei G., Ferrero G.B. (2025). The phenotypes of sickle cell disease: Strategies to aid the identification of undiagnosed patients in the Italian landscape. Ital. J. Pediatr..

[8] Ata F., Rahhal A., Malkawi L., Iqbal P., Khamees I., Alhiyari M., Yousaf Z., Qasim H., Alshurafa A., Sardar S. (2023). Genotypic and Phenotypic Composition of Sickle Cell Disease in the Arab Population—A Systematic Review. Pharmacogenomics Pers. Med..

[9] Taher M., Aminondin S.A., Nasir N.A., Jasmadi N.A., Nizam N.I.N., Shahrul I.S., Susanti D., Khotib J., Faiyazuddin M., Widodo R.T. (2025). Sickle cell disease: Understanding pathophysiology, clinical features and advances in gene therapy approaches. Front. Pharmacol..

[10] Alsalman M., Alkhalifa H., Alkhalifa A., Alsubie M., AlMurayhil N., Althafar A., Albarqi M., Alnaim A., Khan A.S. (2021). Hydroxyurea usage awareness among patients with sickle-cell disease in Saudi Arabia. Health Sci. Rep..

[11] Davis B.A., Allard S., Qureshi A., Porter J.B., Pancham S., Win N., Cho G., Ryan K., British Society for Haematology (2017). Guidelines on red cell transfusion in sickle cell disease Part II: Indications for transfusion. Br. J. Haematol..

[12] Inusa B.P., Atoyebi W., Andemariam B., Hourani J.N., Omert L. (2023). Global burden of transfusion in sickle cell disease. Transfus. Apher. Sci..

[13] Chou S.T., Hendrickson J.E. (2025). How I treat challenging transfusion cases in sickle cell disease. Blood.

[14] Chou S.T., Alsawas M., Fasano R.M., Field J.J., Hendrickson J.E., Howard J., Kameka M., Kwiatkowski J.L., Pirenne F., Shi P.A. (2020). American society of hematology 2020 guidelines for sickle cell disease: Transfusion support. Blood Adv..

[15] Page M.J., McKenzie J.E., Bossuyt P.M., Boutron I., Hoffmann T.C., Mulrow C.D., Shamseer L., Tetzlaff J.M., Akl E.A., Brennan S.E. (2021). The PRISMA 2020 statement: An updated guideline for reporting systematic reviews. BMJ.

[16] Alsalman M., Almaqhawi A., Alnajjar J., Alhassan S., Alshams M., Almogren B., Alhassan R., Almarzoug H., Alobaid Q. Global Alloimmunization Rates in Adults with Sickle Cell Disease Receiving Blood Transfusion: A Systemic Review and Meta-Analysis. PROSPERO 2025 CRD420251167042. https://www.crd.york.ac.uk/PROSPERO/view/CRD420251167042.

[17] Wells G., Shea B., O’Connell D., Peterson J., Welch V., Losos M., Tugwell P. The Newcastle-Ottawa Scale (NOS) for Assessing the Quality of Nonrandomised Studies in Meta-Analyses. https://www.ohri.ca/programs/clinical_epidemiology/oxford.asp.

[18] Sun Q., Karafin M.S., Garrett M.E., Li Y., Ashley-Koch A., Telen M.J. A Genome-Wide Association Study of Alloimmunization in the Topmed OMG-SCD Cohort Identifies a Locus on Chromosome 12 Transfusion. https://pubmed.ncbi.nlm.nih.gov/38966903/.

[19] Leite L.E., Da Silva F.G., Kashima S., Rodrigues E.S., Haddad R. (2023). RHCE and Kell genotyping and alloimmunization profile in patients with sickle cell disease in the Federal District of Brazil. Hematol. Transfus. Cell Ther..

[20] Leal I., Santos T.D.D., Gilli S., Castilho L. (2023). Effects of prophylactic red blood cell (RBC) transfusion with extended antigen matching on alloimmunization in patients with Sickle Cell Disease (SCD). Transfus. Apher. Sci..

[21] AlDawood R. (2022). The Prevalence of Cumulative Alloimmunization in Patients with Sickle Cell Disease at King Fahad University Hospital. https://www.semanticscholar.org/paper/The-prevalence-of-cumulative-alloimmunization-in-at-AlDawood/d2cdaf05e10ba04bdabbbaea71f146434d375732.

[22] Solmaz S., Karacaoğlu P., Gereklioğlu Ç., Asma S., Korur A., Büyükkurt N., Kasar M., Yeral M., Kozanoğlu İ., Boğa C. (2016). Türkiye’ de orak hücre hastalığına sahip hastalarda eritrosit alloimmünizasyonu: Tek merkez geriye dönük kohort çalışması. Cukurova Med. J..

[23] Telen M.J., Afenyi-Annan A., Garrett M.E., Combs M.R., Orringer E.P., Ashley-Koch A.E. (2014). Alloimmunization in sickle cell disease: Changing antibody specificities and association with chronic pain and decreased survival. Transfusion.

[24] Karafin M.S., Field J.J., Gottschall J.L., Denomme G.A. (2015). Barriers to using molecularly typed minority red blood cell donors in support of chronically transfused adult patients with sickle cell disease. Transfusion.

[25] Zanette A.M.D., Gonçalves M.S., Schettini L.V., Aguiar L.M., Bahia R.C.S., Nogueira L.A.V., Brandão C.J.F., Azevedo A.C.N., Aragao L.R., Arruda S.M. (2010). Alloimmunization and clinical profile of sickle cell disease patients from Salvador-Brazil. Ethn. Dis..

[26] Orlina A.R., Unger P.J., Koshy M. (1978). Post-transfusion alloimmunization in patients with sickle cell disease. Am. J. Hematol..

[27] Dinić R., Bujandrić N., Grujić J. (2025). Prevalence and Specificity of Red Blood Cell Alloimmunization: Insights from Transfusion-Dependent Populations in Serbia. Thalass. Rep..

[28] Das S.S., Biswas R.N., Safi M., Zaman R.U. (2021). Alloimmunization to Erythrocyte Antigens in Patients Receiving Multiple Blood Transfusions. Glob. J. Transfus. Med..

[29] El-Beshlawy A., Salama A.A., El-Masry M.R., El Husseiny N.M., Abdelhameed A.M. (2020). A study of red blood cell alloimmunization and autoimmunization among 200 multitransfused Egyptian β thalassemia patients. Sci. Rep..

[30] Al -Asmari B., Baothman A., Almohammadi M., Aljuaid M., Jastaniah W. (2024). Prevalence of red blood cell alloimmunization among pediatric patients with sickle cell disease in Saudi Arabia. J. Pediatr. Hematol. Oncol..

[31] De Oliveira Werneck R.D., Cardoso S.L., Dos S.O.F., Claudio R.L. (2020). Erythrocyte Alloimmunization in Children with Sickle Cell Disease. Int. J. Blood Res. Disord..

[32] Pahuja S., Mandal P. (2024). Alloimmunization and autoimmunization among multitransfused thalassemia and sickle cell disease patients. Pediatr. Hematol. Oncol. J..

[33] Chou S.T., Jackson T., Vege S., Smith-Whitley K., Friedman D.F., Westhoff C.M. (2013). High prevalence of red blood cell alloimmunization in sickle cell disease despite transfusion from Rh-matched minority donors. Blood.

[34] Pirenne F., Pondarré C. (2023). Alloimmunization and hyperhemolysis in sickle cell disease. Hematology.

[35] Thomas T.A., Qiu A., Kim C.Y., Gordy D.E., Miller A., Tredicine M., Dzieciatkowska M., Zotti F.D., Hod E.A., D’Alessandro A. (2023). Reticulocytes in donor blood units enhance red blood cell alloimmunization. Haematologica.

[36] Sins J.W.R., Biemond B.J., van den Bersselaar S.M., Heijboer H., Rijneveld A.W., Cnossen M.H., Kerkhoffs J.H., van Meurs A.H., von Ronnen F., Zalpuri S. (2016). Early occurrence of red blood cell alloimmunization in patients with sickle cell disease. Am. J. Hematol..

[37] Idris I.M., Botchwey E.A., Hyacinth H.I. (2022). Sickle cell disease as an accelerated aging syndrome. Exp. Biol. Med..

[38] Jagadeeswaran R., Rivers A. (2017). Evolving treatment paradigms in sickle cell disease. Hematology.

[39] Han H., Hensch L., Tubman V.N. (2021). Indications for transfusion in the management of sickle cell disease. Hematology.

[40] Yazdanbakhsh K., Ware R.E., Noizat-Pirenne F. (2012). Red blood cell alloimmunization in sickle cell disease: Pathophysiology, risk factors, and transfusion management. Blood.

[41] Wolf J., Blais-Normandin I., Bathla A., Keshavarz H., Chou S.T., Al-Riyami A.Z., Josephson C.D., Massey E., Hume H.A., Pendergrast J. (2024). Red cell specifications for blood group matching in patients with hemoglobinopathies: An updated systematic review and clinical practice guideline from the International Collaboration for Transfusion Medicine Guidelines. Br. J. Haematol..

