# Supplementary material for: Global Prevalence of Alloimmunization in Adults with Sickle Cell Disease Receiving Red Blood Cell Transfusions: A Systematic Review and Meta-Analysis
Source: J Clin Med. 2026 May 15;15(10):3828. doi: 10.3390/jcm15103828 (PMC13207003; doi:10.3390/jcm15103828)
Supplement: Supplementary file 1 [file jcm-15-03828-s001.zip › jcm-4096258-supplementary.pdf]

Table S1. PRISMA-2020 checklist.

Section and Topic	Item #	Checklist item	Location where item is reported
**TITLE**			
Title	1	Identify the report as a systematic review.	P1 lines 1-4
**ABSTRACT**			
Abstract	2	See the PRISMA 2020 for Abstracts checklist.	P1 lines 16-43
**INTRODUCTION**			
Rationale	3	Describe the rationale for the review in the context of existing knowledge.	P2 lines 77-83
Objectives	4	Provide an explicit statement of the objective(s) or question(s) the review addresses.	P2 lines 81-83
**METHODS**			
Eligibility criteria	5	Specify the inclusion and exclusion criteria for the review and how studies were grouped for the syntheses.	P4 lines 113-128
Information sources	6	Specify all databases, registers, websites, organisations, reference lists and other sources searched or consulted to identify studies. Specify the date when each source was last searched or consulted.	P3 lines 93-95
Search strategy	7	Present the full search strategies for all databases, registers and websites, including any filters and limits used.	P3-4 lines 87-111
Selection process	8	Specify the methods used to decide whether a study met the inclusion criteria of the review, including how many reviewers screened each record and each report retrieved, whether they worked independently, and if applicable, details of automation tools used in the process.	P3 lines 107-128
Data collection process	9	Specify the methods used to collect data from reports, including how many reviewers collected data from each report, whether they worked independently, any processes for obtaining or confirming data from study investigators, and if applicable, details of automation tools used in the process.	P3-4 lines 93-111
Data items	10a	List and define all outcomes for which data were sought. Specify whether all results that were compatible with each outcome domain in each study were sought (e.g. for all measures, time points, analyses), and if not, the methods used to decide which results to collect.	P4-5 lines 138-151
	10b	List and define all other variables for which data were sought (e.g. participant and intervention characteristics, funding sources). Describe any assumptions made about any missing or unclear information.	P4-5 lines 131-151
Study risk of bias assessment	11	Specify the methods used to assess risk of bias in the included studies, including details of the tool(s) used, how many reviewers assessed each study and whether they worked independently, and if applicable, details of automation tools used in the process.	P5 lines 153-170
Effect measures	12	Specify for each outcome the effect measure(s) (e.g. risk ratio, mean difference) used in the synthesis or presentation of results.	P5-6 lines 172-198
Synthesis methods	13a	Describe the processes used to decide which studies were eligible for each synthesis (e.g. tabulating the study intervention characteristics and comparing against the planned groups for each synthesis (item #5)).	P4 lines 113-128
	13b	Describe any methods required to prepare the data for presentation or synthesis, such as handling of missing summary statistics, or data conversions.	P6 lines 179-183 196-198

Section and Topic	Item #	Checklist item	Location where item is reported
	13c	Describe any methods used to tabulate or visually display results of individual studies and syntheses.	P8 lines 270-274
	13d	Describe any methods used to synthesize results and provide a rationale for the choice(s). If meta-analysis was performed, describe the model(s), method(s) to identify the presence and extent of statistical heterogeneity, and software package(s) used.	P6-7-8 lines 185-192 200-217 268-274
	13e	Describe any methods used to explore possible causes of heterogeneity among study results (e.g. subgroup analysis, meta-regression).	P7 lines 217-247
	13f	Describe any sensitivity analyses conducted to assess robustness of the synthesized results.	None
Reporting bias assessment	14	Describe any methods used to assess risk of bias due to missing results in a synthesis (arising from reporting biases).	P5 lines 251-266
Certainty assessment	15	Describe any methods used to assess certainty (or confidence) in the body of evidence for an outcome.	None
**RESULTS**			
Study selection	16a	Describe the results of the search and selection process, from the number of records identified in the search to the number of studies included in the review, ideally using a flow diagram.	P8 lines 277-283
	16b	Cite studies that might appear to meet the inclusion criteria, but which were excluded, and explain why they were excluded.	P8 lines 277-283
Study characteristics	17	Cite each included study and present its characteristics.	P9-10 lines 287-299
Risk of bias in studies	18	Present assessments of risk of bias for each included study.	P18-19 lines 432-449
Results of individual studies	19	For all outcomes, present, for each study: (a) summary statistics for each group (where appropriate) and (b) an effect estimate and its precision (e.g. confidence/credible interval), ideally using structured tables or plots.	P9-15 lines 296-299 310-313 348-358 370-371 386-392
Results of syntheses	20a	For each synthesis, briefly summarise the characteristics and risk of bias among contributing studies.	P8-9 lines 277-299
	20b	Present results of all statistical syntheses conducted. If meta-analysis was done, present for each the summary estimate and its precision (e.g. confidence/credible interval) and measures of statistical heterogeneity. If comparing groups, describe the direction of the effect.	P11 lines 326-347
	20c	Present results of all investigations of possible causes of heterogeneity among study results.	P11 lines 326-347
	20d	Present results of all sensitivity analyses conducted to assess the robustness of the synthesized results.	None
Reporting biases	21	Present assessments of risk of bias due to missing results (arising from reporting biases) for each synthesis assessed.	Not assessed
Certainty of	22	Present assessments of certainty (or confidence) in the body of evidence for each outcome assessed.	Not

Section and Topic	Item #	Checklist item	Location where item is reported
evidence			assessed
**DISCUSSION**			
Discussion	23a	Provide a general interpretation of the results in the context of other evidence.	P19 lines 455-457
	23b	Discuss any limitations of the evidence included in the review.	P22 lines 544-549
	23c	Discuss any limitations of the review processes used.	P22 lines 542-549
	23d	Discuss implications of the results for practice, policy, and future research.	P22 lines 512-536
**OTHER INFORMATION**			
Registration and protocol	24a	Provide registration information for the review, including register name and registration number, or state that the review was not registered.	P1 lines 21-22
	24b	Indicate where the review protocol can be accessed, or state that a protocol was not prepared.	P1 lines 21-22
	24c	Describe and explain any amendments to information provided at registration or in the protocol.	P1 lines 21-22
Support	25	Describe sources of financial or non-financial support for the review, and the role of the funders or sponsors in the review.	P22 lines 560-562
Competing interests	26	Declare any competing interests of review authors.	P22 lines 575-576
Availability of data, code and other materials	27	Report which of the following are publicly available and where they can be found: template data collection forms; data extracted from included studies; data used for all analyses; analytic code; any other materials used in the review.	P22 lines 557-558 577-579

From: Page MJ, McKenzie JE, Bossuyt PM, Boutron I, Hoffmann TC, Mulrow CD, et al. The PRISMA 2020 statement: an updated guideline for reporting systematic reviews. BMJ 2021;372:n71. doi: 10.1136/bmj.n71. This work is licensed under CC BY 4.0. To view a copy of this license, visit https://creativecommons.org/licenses/by/4.0/

Table S2. Search strategy used in each of the databases.

Database	Search Strategy Used
PubMed	(alloantibodies OR allo-antibodies OR isoimmunization OR iso-immunization OR isoantibodies OR red cell alloantibodies OR unexpected alloantibodies OR irregular erythrocyte antibodies OR erythrocyte alloantibodies OR alloimmunisation OR alloimmunization) AND (sickle cell anemia OR sickle cell anaemia OR HbSS OR HbSC OR HbSβ-thalassemia OR "hemoglobin S disease" OR "sickle cell disorders" OR HbS disease OR sickling disorder due to hemoglobin S OR sickle cell disease OR SCD) AND (blood transfusion OR blood transfusions OR transfusion OR "red blood cell transfusion" OR RBC transfusion
PubMed Updated 7 November	(alloantibodies OR allo-antibodies OR isoimmunization OR iso-immunization OR isoantibodies OR red cell alloantibodies OR unexpected alloantibodies OR irregular erythrocyte antibodies OR erythrocyte alloantibodies OR alloimmunisation OR alloimmunization) AND (sickle cell anemia OR sickle cell anaemia OR

2024	HbSS OR HbSC OR HbSβ-thalassemia OR "hemoglobin S disease" OR "sickle cell disorders" OR HbS disease OR sickling disorder due to hemoglobin S OR sickle cell disease OR SCD) AND (blood transfusion OR blood transfusions OR transfusion OR "red blood cell transfusion" OR RBC transfusion)<br>=72
Embase	Embase <1974 to 2024 Week 44><br><br>1 alloantibodies.mp. or alloantibody/ 11978<br>2 allo-antibodies.mp. 526<br>3 isoimmunization.mp. 2208<br>4 iso-immunization.mp. 74<br>5 isoantibodies.mp. 251<br>6 red cell alloantibodies.mp. 289<br>7 unexpected alloantibodies.mp. 31<br>8 irregular erythrocyte antibodies.mp. 52<br>9 erythrocyte alloantibodies.mp. 61<br>10 alloimmunisation.mp. 805<br>11 alloimmunization.mp. 7912<br>12 1 or 2 or 3 or 4 or 5 or 6 or 7 or 8 or 9 or 10 or 11 20102<br>13 sickle cell anemia.mp. 46761<br>14 HbSS.mp. 4226<br>15 hemoglobin S disease.mp. 35<br>16 sickle cell disorders.mp. 284<br>17 HbS disease.mp. 25<br>18 sickling disorder due to hemoglobin S.mp. 0<br>19 sickle cell disease.mp. 32344<br>20 SCD.mp. 34479<br>21 13 or 14 or 15 or 16 or 17 or 18 or 19 or 20 69794<br>22 blood transfusion.mp. 186124<br>23 blood transfusions.mp. 25527<br>24 transfusion.mp. 309523<br>25 red blood cell transfusion.mp. 5569<br>26 22 or 23 or 24 or 25 313288<br>27 12 and 21 and 26 1276
Embase Updated 7 November 2024	Embase <1974 to 2026 Week 15><br><br>1 alloantibodies.mp. or alloantibody/ 12781<br>2 allo-antibodies.mp. 572<br>3 isoimmunization.mp. 2281<br>4 iso-immunization.mp. 77<br>5 isoantibodies.mp. 266<br>6 red cell alloantibodies.mp. 322<br>7 unexpected alloantibodies.mp. 33<br>8 irregular erythrocyte antibodies.mp. 56<br>9 erythrocyte alloantibodies.mp. 73<br>10 alloimmunisation.mp. 931<br>11 alloimmunization.mp. 8853<br>12 1 or 2 or 3 or 4 or 5 or 6 or 7 or 8 or 9 or 10 or 11 21632<br>13 sickle cell anemia.mp. 53306<br>14 HbSS.mp. 4810<br>15 hemoglobin S disease.mp. 46<br>16 sickle cell disorders.mp. 326<br>17 HbS disease.mp. 35<br>18 sickling disorder due to hemoglobin S.mp. 13<br>19 sickle cell disease.mp. 37394<br>20 SCD.mp. 41128

	21 13 or 14 or 15 or 16 or 17 or 18 or 19 or 20 79616<br>22 blood transfusion.mp. 204006<br>23 blood transfusions.mp. 29214<br>24 transfusion.mp. 341003<br>25 red blood cell transfusion.mp. 6454<br>26 22 or 23 or 24 or 25 344914<br>27 12 and 21 and 26 1564<br>28 limit 27 to yr="2024 -Current" 209
Scopus	(alloantibodies OR allo-antibodies OR isoimmunization OR iso-immunization OR isoantibodies OR red cell alloantibodies OR unexpected alloantibodies OR irregular erythrocyte antibodies OR erythrocyte alloantibodies OR alloimmunisation OR alloimmunization) AND (sickle cell anemia OR sickle cell anaemia OR HbSS OR HbSC OR HbSβ-thalassemia OR "hemoglobin S disease" OR "sickle cell disorders" OR HbS disease OR sickling disorder due to hemoglobin S OR sickle cell disease OR SCD) AND (blood transfusion OR blood transfusions OR transfusion OR "red blood cell transfusion" OR RBC transfusion)
Scopus Updated 7 November 2024	(alloantibodies OR allo-antibodies OR isoimmunization OR iso-immunization OR isoantibodies OR red cell alloantibodies OR unexpected alloantibodies OR irregular erythrocyte antibodies OR erythrocyte alloantibodies OR alloimmunisation OR alloimmunization) AND (sickle cell anemia OR sickle cell anaemia OR HbSS OR HbSC OR HbSβ-thalassemia OR "hemoglobin S disease" OR "sickle cell disorders" OR HbS disease OR sickling disorder due to hemoglobin S OR sickle cell disease OR SCD) AND (blood transfusion OR blood transfusions OR transfusion OR "red blood cell transfusion" OR RBC transfusion)
Web of science	(alloantibodies OR allo-antibodies OR isoimmunization OR iso-immunization OR isoantibodies OR red cell alloantibodies OR unexpected alloantibodies OR irregular erythrocyte antibodies OR erythrocyte alloantibodies OR alloimmunisation OR alloimmunization) AND (sickle cell anemia OR sickle cell anaemia OR HbSS OR HbSC OR HbSβ-thalassemia OR "hemoglobin S disease" OR "sickle cell disorders" OR HbS disease OR sickling disorder due to hemoglobin S OR sickle cell disease OR SCD) AND (blood transfusion OR blood transfusions OR transfusion OR "red blood cell transfusion" OR RBC transfusion)
Web of science Updated 7 November 2024	alloantibodies OR allo-antibodies OR isoimmunization OR iso-immunization OR isoantibodies OR red cell alloantibodies OR unexpected alloantibodies OR irregular erythrocyte antibodies OR erythrocyte alloantibodies OR alloimmunisation OR alloimmunization) AND (sickle cell anemia OR sickle cell anaemia OR HbSS OR HbSC OR HbSβ-thalassemia OR "hemoglobin S disease" OR "sickle cell disorders" OR HbS disease OR sickling disorder due to hemoglobin S OR sickle cell disease OR SCD) AND (blood transfusion OR blood transfusions OR transfusion OR "red blood cell transfusion" OR RBC transfusion)

Table S3. Risk of bias assessment.

Supplementary Table 1: Risk of Bias Assessment Using Newcastle-Ottawa Scale.

Study	Study Design	Selection (0-4★)	Comparability (0-2★)	Outcome Assessment (0-3★)	Total NOS Score	Quality Rating	Major Limitations	Overall Risk of Bias	Justification
Sun et al. 2024	Retrospective	★★★★ (4/4)	★★ (2/2)	★☆☆ (1/3)	7/9	Good	Retrospective design; self-reported transfusion history; relatively small sample for GWAS	Moderate	Strong ascertainment and adjustment for covariates, but limited by retrospective design and incomplete follow-up data
Leite et al. 2024	Cross-sectional	★★★☆ (3/4)	☆☆ (0/2)	★★☆ (2/3)	5/9	Fair	Small sample size (N=77); no multivariable analysis; transfusion history not reported; cross-sectional design limits causal inference	Moderate	Validated detection method but limited sample size and lack of adjustment for confounders
Leal et al. 2023	Retrospective	★★★★ (4/4)	☆☆ (0/2)	★★★ (3/3)	7/9	Good	Retrospective design; no multivariable analysis despite long follow-up	Low	Excellent follow-up duration (20 years) with gold-standard genotyping method; good sample retention
AlDawood et al. 2022	Retrospective	★★★★ (4/4)	★★ (2/2)	★★☆ (2/3)	8/9	Good	Retrospective design; transfusion history outside study institution untraceable	Low	Largest sample size with multivariable analysis and validated methods; well-controlled for confounders

Solmaz et al. 2016	Retrospective	★★★☆ (3/4)	☆☆ (0/2)	★☆☆ (1/3)	4/9	Fair	High exclusion rate (42%, 160/376 patients); missing antibody specificity data; retrospective design; no multivariable analysis	High	Substantial attrition bias due to high exclusion rate; incomplete outcome data limits interpretation
Telen et al. 2015	Retrospective	★★★☆ (3/4)	★★ (2/2)	★☆☆ (1/3)	6/9	Fair	Retrospective design; self-reported transfusion history; follow-up duration not reported	Moderate	Good sample size with multivariable analysis, but limited by self-report and unclear follow-up
Karafin et al. 2015	Retrospective	★★☆☆ (2/4)	☆☆ (0/2)	★★★ (3/3)	5/9	Fair	Small sample size (N=54); pre-2010 transfusion history undefined; no multivariable analysis	Moderate	Excellent detection methods but limited by small sample and incomplete historical data
Zanette et al. 2010	Cross-sectional	★☆☆☆ (1/4)	☆☆ (0/2)	☆☆☆ (0/3)	1/9	Poor	Detection method not reported; small sample size; potential underestimation of transfusion burden; no multivariable analysis; cross-sectional design	High	Multiple methodological concerns including unreported detection method and no adjustment for confounders
Orlina et al. 1978	Retrospective	★☆☆☆ (1/4)	☆☆ (0/2)	★★☆ (2/3)	3/9	Fair	Historical study (1978); small sample (N=50); pre-study transfusion history unknown; descriptive analysis only; limited reporting standards for era	Moderate-High	Historical study with methodological limitations typical of era; provides useful early data but multiple biases

Abbreviations: *NOS, Newcastle-Ottawa Scale; GWAS, genome-wide association study; N, sample size; ★, criterion met (star awarded); ☆, criterion not met (no star).*
